# RETRACTION: Evaluation of In Vivo Wound‐Healing and Anti‐Inflammatory Activities of Solvent Fractions of Fruits of *Argemone mexicana* L. (Papaveraceae)

**DOI:** 10.1155/ecam/9824261

**Published:** 2026-02-07

**Authors:** Evidence-Based Complementary and Alternative Medicine

RETRACTION: T. M. Ayele, E. C. Abebe, Z. T. Muche, et al., “Evaluation of In Vivo Wound‐Healing and Anti‐Inflammatory Activities of Solvent Fractions of Fruits of *Argemone mexicana* L. (Papaveraceae),” *Evidence-Based Complementary and Alternative Medicine*, (2022): 6154560, https://doi.org/10.1155/2022/6154560.

The above article, published online on 22 November 2022 in Wiley Online Library (https://wileyonlinelibrary.com), has been retracted by agreement between the journal Editor‐in‐Chief, Anshu Agrawal, and John Wiley & Sons Ltd. The retraction has been agreed due to figure concerns that have been raised on PubPeer [1]. On further investigation, the below inconsistencies with Figures 3 and 4 have been identified:

• In Figure 3, the Day 9 MEBO and Day 9 10% n‐BF images appear similar.
• In Figure 3, the Day 9 10% n‐BF image and the Day 15 5% n‐BF image appear similar.
• In Figure 3, the Day 18 5% n‐BF image and the Day 15 10% n‐BF image appear similar.
• In Figure 4, there are repeated regions between panels B and F.
• In Figure 4, panel G appears to be the same as panel A of Figure 6 in [2].
• In Figure 4, panel D appears to be the same as panel D of Figure 6 in [2].
• In Figure 4, panel H appears to be the same as panel B of Figure 6 in [2].
• In Figure 4, panel B appears to be the same as top left panel of Figure 9 in [2].
• In Figure 4, panel E appears to be the same as bottom right panel of Figure 9 in [2].
• In Figure 4, panel A appears to be the same as the bottom left panel of Figure 9 in [2].
• In Figure 4, panel F appears to be the same as the top right panel of Figure 9 in [2].

The authors did not provide a response to the concerns or retraction, and the Chief Editor has confirmed the retraction due to the unreliability of the data.
